# Suicide during Perinatal Period: Epidemiology, Risk Factors, and Clinical Correlates

**DOI:** 10.3389/fpsyt.2016.00138

**Published:** 2016-08-12

**Authors:** Laura Orsolini, Alessandro Valchera, Roberta Vecchiotti, Carmine Tomasetti, Felice Iasevoli, Michele Fornaro, Domenico De Berardis, Giampaolo Perna, Maurizio Pompili, Cesario Bellantuono

**Affiliations:** ^1^School of Life and Medical Sciences, University of Hertfordshire, Hatfield, UK; ^2^Villa San Giuseppe Hospital, Hermanas Hospitalarias, Ascoli Piceno, Italy; ^3^Polyedra Research Group, Teramo, Italy; ^4^Department of Psychiatry and Neuropsychology, University of Maastricht, Maastricht, Netherlands; ^5^Department of Mental Health ASL Teramo, Psychiatric Service of Diagnosis and Treatment, NHS, Hospital “Maria SS dello Splendore”, Giulianova, Italy; ^6^Laboratory of Molecular and Translational Psychiatry, Department of Neuroscience, Reproductive and Odontostomatological Sciences, University of Naples “Federico II”, Napoli, Italy; ^7^New York Psychiatric Institute, Columbia University, New York City, NY, USA; ^8^Department of Mental Health ASL Teramo, Psychiatric Service of Diagnosis and Treatment, NHS, Hospital “G. Mazzini”, Teramo, Italy; ^9^Department of Neuroscience and Imaging, University “G. d’Annunzio”, Chieti, Italy; ^10^Department of Clinical Neurosciences, Hermanas Hospitalarias, FoRiPsi, Villa San Benedetto Menni, Albese con Cassano, Como, Italy; ^11^Department of Psychiatry and Behavioral Sciences, Leonard Miller School of Medicine, University of Miami, Coral Gables, FL, USA; ^12^Department of Psychiatry, Sant’Andrea Hospital, Rome, Italy; ^13^DEGRA Clinic, Verona-Rimini-Ancona, Italy

**Keywords:** suicide, suicidal ideation, suicide attempt, pregnancy, perinatal period, puerperium, postpartum

## Abstract

Perinatal period may pose a great challenge for the clinical management and treatment of psychiatric disorders in women. In fact, several mental illnesses can arise during pregnancy and/or following childbirth. Suicide has been considered a relatively rare event during the perinatal period. However, in some mental disorders (i.e., postpartum depression, bipolar disorder, postpartum psychosis, etc.) have been reported a higher risk of suicidal ideation, suicide attempt, or suicide. Therefore, a complete screening of mothers’ mental health should also take into account thoughts of suicide and thoughts about harming infants as well. Clinicians should carefully monitor and early identify related clinical manifestations, potential risk factors, and alarm symptoms related to suicide. The present paper aims at providing a focused review about epidemiological data, risk factors, and an overview about the main clinical correlates associated with the suicidal behavior during the pregnancy and postpartum period. Practical recommendations have been provided as well.

## Introduction

Suicide was worldwide ranked as the 14th leading cause of mortality and morbidity, and it is expected to increase by 50%, becoming the 12th leading cause of mortality by year 2030 (1). Suicide represents a major public health problem, with more than 1,000,000 suicides worldwide (2). A preexisting vulnerability, such as a family history of suicide, impulsivity, and previous and/or current psychiatric diagnoses, may be precipitating risk factors for suicidal ideation (SI) and behavior (3, 4). Specifically, major depressive disorder (MDD) and other affective disorders may represent strong risk factors for suicide (2). Furthermore, SI and a history of personal suicidal behavior are among the most salient short- and long-term risk factors for suicide (2). The National Institute of Mental Health (NIMH) Developing Centers for Intervention and Prevention of Suicide defined the SI as the wish to die, thoughts of killing oneself, and the intent to kill oneself (5). Thoughts of killing oneself are thoughts, beliefs, images, voices, or other cognitions about intentionally ending one’s own life (suicide) and may include the intent to act on such thoughts (2). SI or thoughts may be often associated with suicide attempts and completions (6, 7).

The identification of SI and behavior, as well as high-risk individuals, requires an adequate suicide screening assessment (2), particularly during the perinatal period. Generally, peripartum (including conception, pregnancy, and postpartum) may be a period of considerable vulnerability to MDD and affective disorders as well as it is frequently associated with the onset and/or recrudescence of a psychiatric illness (8, 9). Overall, approximately 10–15% of newly delivered women experience a major depressive episode; while around 50% of women with a previous mood disorder and 70% with a family history of postpartum psychosis will develop a relapse and/recrudescence following a subsequent delivery (10).

Although suicides and suicidal attempts occur at a lower rate during pregnancy and the postpartum period than in general population (10), the prevalence of SI or thoughts ranges from 5 to 14% (9, 11, 12) and, sometimes, it may result in a suicide attempts and completions (6, 11). In fact, perinatal suicidality, which comprises completed suicides, suicide attempts, SI, and thoughts of self-harm, is nowadays considered one of the leading causes of maternal mortality in the first 12 months postpartum (11, 13–15). Furthermore, it has been documented that women reporting SI and thoughts during pregnancy or during the postpartum period had higher odds for developing postpartum depression (16, 17).

The increasing need of a careful assessment and screening of pregnant and nursing women’s mental health should also take into account SI, thoughts of suicide, and thoughts about harming infants as well. Clinicians should carefully monitor and early identify related clinical manifestations, potential risk factors, and alarm symptoms related to suicide. Therefore, the present paper aims at providing a focused review about epidemiological data, risk and protective factors, and an overview about the main clinical correlates associated with the suicidal behavior during the pregnancy and postpartum period.

## Materials and Methods

The present review was carried out in accordance to the methods recommended by the Preferred Reporting Items for Systematic Reviews and Meta-Analyses (PRISMA) guidelines (18). Studies were identified searching the electronic databases MEDLINE, Embase, PsycINFO, and the Cochrane Library. A combined search strategy of free text terms and exploded MESH headings for the topics of suicide and perinatal period as following: ((*suicide* [Title/Abstract] OR *suicide attempt* [Title/Abstract] OR *suicidal ideation* [Title/Abstract]) OR *puerperium* [Title/Abstract]). (“*suicide*” [MeSH Terms] OR “*suicide”* [Title/Abstract]) AND (“*pregnancy*” [MeSH Terms] OR “*pregnancy*” [Title/Abstract]) OR (“*postpartum period*” [MeSH Terms] OR “*postpartum*” [Title/Abstract] OR “*postpartum period*” [Title/Abstract]) OR Studies published in English through March 20, 2015 were included. In addition, further studies were retrieved from reference listing of relevant articles and consultation with experts in the field and or manual search. Identified studies were independently reviewed for eligibility by two authors (Laura Orsolini and Alessandro Valchera) in a two-step based process; a first screening was performed based on title and abstract, while full texts were retrieved for the second screening. At both stages, disagreements by reviewers were resolved by consensus. Data were extracted by two authors (Laura Orsolini and Alessandro Valchera) and supervised by a third author (Cesario Bellantuono) using an *ad hoc* developed data extraction spreadsheet. With the initial set of keywords, 1,567 studies were identified. Of these, 219 were excluded because they were not in English, 1,219 were excluded because they were either duplicated or not consistent with the aims of the review. Of these remaining, 129 were relevant studies. Data were then analyzed according to the epidemiology, risk factors, and clinical correlates.

## Results

### Epidemiology

A recent 15-year (1997–2012) retrospective study, from the UK National Confidential Inquiry that evaluated all suicides by people who had been in contact with psychiatric services, compared suicides among perinatal and non-perinatal women. Findings reported a suicide in perinatal period in 2% of women aged 16–50 years and in 4% among women aged 20–35 years (19). A register-based cohort Danish study reported, among women with severe postpartum psychiatric disorders, a suicide risk drastically increased when compared with mother with no psychiatric history, by suggesting a strong correlation between perinatal suicidality and psychiatric conditions (20). A retrospective cohort study carried out on perinatal women at 24–28 weeks of gestation and 6 weeks postpartum reported an SI in 3.8% of the women screened with the Edinburgh Postnatal Depression Scale (EPDS), with only 1.1% with a high risk for suicide (i.e., with plan, intent, and access to suicidal means) (21). A register-based Swedish study reported a maternal suicide ratio of 3.7 per 100,000 live births for the period 1980–2007, being higher among women born in low-income countries (22). An audit cohort study evaluated 225 women afferent to a UK perinatal mental health team over a 12-month period. Suicide attempts occurred among women with a previous postpartum depression in 24–49% (10). A prospective study evaluating thoughts of self-harm and SI during the postpartum period among women with mood disorders reported thoughts of self-harm and SI, respectively, in 16.97 and 6.16% of the sample, during the 1-year postpartum period (23). A US community-based study reported a 14-day prevalence of antenatal SI of 2.7% (24). Findings from PND-ReScU reported a rate ranging 6.9–12% of suicidality during pregnancy, while a rate of 4.3–8.6% during the postpartum period, depending on the assessment tools (25). Women who had major or minor depressive episode during pregnancy showed a prevalence of suicidality of 26.4 and 34.1%, while it was 18.4–30.6% during the postpartum period (25). A prospective cohort study evaluated the prevalence of SI as measured by the EPDS in a primary care population of women at 6–8 weeks postpartum screened for postnatal depressive symptoms. The authors reported that 4% of women in the community had SI occurring sometimes or quite often, while 9% reported any SI (26). Suicide attempts appear to be more frequent in the 1st and 12th months after delivery (27).

Furthermore, according to the NSW Australian Department of Health evaluating a 6-year time period, 73% of suicides by women within 1 year of birth were conducted by violent means (i.e., jumping from high place, lying in front of moving objects, gunshot, strangulation, and suffocation) (14, 22).

### Risk Factors

Suicides in the perinatal period appear to be more likely occurring among women who are less likely to be receiving any active treatment at the time of death, younger maternal age, unpartnered relationship status, unplanned pregnancy, non-Caucasian race, with shorter illness duration, preexisting, and/or current psychiatric diagnosis (9, 19, 21, 24, 26, 28–34). Regarding the delivery, severe vaginal laceration was positively correlated with SI risk, while planned cesarean delivery was negatively associated (21). Furthermore, experiencing an intimate partner violence, including emotional abuse, physical, and/or sexual violence, seems to be more likely associated with suicidal thoughts during pregnancy and after childbirth (29, 35–37). A cross-sectional study evaluating a sample of pregnant teenagers found a significant association of suicidality with the 18- to 19-year-old subgroup, low education, prior abortion, physical abuse within the last 12 months, and current psychiatric disorders (38). Women who did not desire or had mixed feelings about being pregnant experienced a higher risk of SI (9). A case–control study comparing 520 women who were hospitalized for a postpartum suicide attempt with 2,204 control women who were not hospitalized for a postpartum suicide attempt concluded that maternal complications (i.e., labor, delivery complications, cesarean delivery, etc.) and adverse infant outcomes (i.e., preterm delivery, low birth weight, congenital malformations, etc.) were not associated with a hospitalization for a suicide attempt within 1 year after delivery (27). A cross-sectional analyses carried out on 234 pregnant women enrolled in a prospective cohort study in Brazil reported a higher likelihood of suicide risk among women with higher arachidonic acid and adrenic acid levels (39). Women who have had a postpartum psychiatric admission have a 70 times greater risk of suicide in their first postpartum year (40, 41).

All risk factors are summarized in Table 1.

**Table 1:** **Risk factors for perinatal suicidality**.

Risk factors for perinatal suicidality	
Individual risk factors	Younger age (9, 19, 21, 24, 26, 28–34) Being unmarried (9, 19, 21, 24, 26, 28–34) Personal and/or family history of psychiatric disorders (9, 19, 21, 24, 25, 28–34, 42–46) Personal and/or family history of suicidal attempt or suicidal ideation (9, 19, 21, 24, 25, 28–34, 42–46)
Socioeconomical risk factors	Family conflict (35–37) Exposure to (domestic) physical/psychological violence (35–37) Loneliness and lack of social/family/partner support (35–37) Partner who rejected paternity (35–37)
Environmental risk factors	Social and gender inequalities (28, 37) Social and racial discrimination (28, 37) Belonging to an ethnic or religious minority (28) Crowded or inadequate housing (24, 28, 37) Living in rural areas (37) Exposure to disaster, conflict, war (24)
Gestational risk factors	Unwanted/unintended pregnancy (9, 17) Nulliparity (32)
Clinical risk factors	Previous history of psychiatric disorders (9, 19, 21, 24, 25, 28–34, 42–46) Previous history of suicidal attempt or suicidal ideation (9, 19, 21, 24, 25, 28–34, 42–46) Psychiatric comorbidity (9, 19, 21, 24, 25, 28–34, 42–46) Shorter illness duration (9, 19, 21, 24, 25, 28–34, 42–46) Psychological symptoms (i.e., premenstrual irritability, perceived pregnancy complications, negative attitude toward the pregnancy, anxiety about birth, distancing pattern of coping, etc.) (9, 21)

### Clinical Correlates

Women who reported antenatal SI were more likely to experience comorbid antenatal MMD and antenatal panic disorder (24). In particular, preexisting and/or current psychiatric diagnoses represent strong risk factors, being MDD, comorbid anxiety disorders, sleep disturbances, or a substance and/or alcohol use disorder the most frequently identified (9, 19, 21, 24, 25, 28–34). A positive correlation with antenatal depression, lifetime bipolar disorder, and any current anxiety disorder as well as Beck Depression Inventory (BDI) scores ≥15 and EPDS scores ≥11 has been reported (42). Furthermore, poor subjective sleep quality was associated with increased odds of SI as well (43). Women with a dysphoric-dysregulated temperament are more likely to be at risk of suicide after delivering (44). Generally, an abrupt discontinuation of psychotropic medications during pregnancy has been linked to a higher risk of maternal suicidality (45). A history of a previous suicide attempts represents a strong risk for perinatal suicidality (46).

## Discussion

Although the incidence of suicide among women who have given birth during the past 12 months is lower than that of women who have not given birth, suicide still remains one of the most common leading causes of maternal death during the 1 year following delivery (22, 40, 47). Perinatal suicide occurs mainly through more violent methods compared to suicide in non-pregnant women (11) and at a higher rate among women with a previous or current mental illness (11, 16). Despite very limited and contrasting studies, SI and suicides appear to be more likely to occur during pregnancy rather than postpartum (25).

However, the most important cognitive risk factor to consider in determining a risk of making a suicide attempt or dying by suicide is SI. SI predicts later suicidal behavior (including suicide and suicidal attempt) (48).

Therefore, a preventive and careful assessment, screening, and identification for SI should be included during the perinatal period (Table 2). However, a specific screening for suicide and SI is almost rare, mainly due to time constraints in prenatal care clinics, the lack of proper screening tools, and the missing collaboration between gynecologists/pediatricians and Mental Health’s professionals (24). In fact, SI is usually assessed along with depression screening rather than with specifically designed tools (44, 49–51), such as the Scale for Suicide Ideation (SSRI), the Columbia-Suicide Severity Rating Scale (C-SSRS), and the Suicide Probability Scale (SPS) (2). Among the most widely used antepartum depression screening instruments, the Patient Health Questionnaire-9 (PHQ-9) (52) and the EPDS (51) represent easy tools that may be helpful both in primary care and community maternity services to screen perinatal depressive and anxiety disorders as well as SI (53, 54). PHQ-9 is a 9-item, depression screening scale (52). Item 9 (“*thoughts that you would be better off dead, or of hurting yourself*”) of the PHQ-9, which assesses SI, has been correlated with item 10 of the EPDS (55). Subjects who answered “*several days*,” “*more than half the days*,” or “*nearly every day*” at item 9 were evaluated at risk of SI. While the EPDS (51), a 10-item self-report questionnaire, is usually administered to screen for postnatal depression. SI was defined as an answer of “*sometimes*” or “*yes, quite often*” to question 10 of the EPDS “*The thought of harming myself has occurred to me*.” Further assessment tools may comprise the BDI at item 9 (50) and the Hamilton Rating Scale for Depression (HRSD) at item 3 (49, 56). The BDI is a 21-item self-rated depression scale widely used to screen for depression. The HRSD is a clinician-rated scale. Girardi et al. (44) included the Suicidal History Self-rating Screening Scale in their perinatal assessment of women at risk of suicide.

**Table 2:** **Risk assessment**.

Risk factors for perinatal suicidality	
Clinical risk assessment (9, 19, 21, 24, 25, 28–34, 42–46, 49–51)	Current presentation of suicidality Psychiatric disorders History of current illness Current medications Psychosocial environment Current alcohol and/or drug use Individual strengths and vulnerabilities
SI risk assessment (9, 19, 21, 24, 25, 28–34, 42–46, 49–51)	Nature Timing Persistence of the desire Intent of SI
Suicide plan risk assessment (44, 53–55)	Lethality of the plan The level of detail and violence The level of access to means (e.g., weapon or store of medication)
Current or previous suicidal attempt risk assessment (9, 19, 21, 24, 25, 28–34, 42–46, 49–51)	Timing Intent Method Consequences of the suicidal attempt
Estimating suicide risk (24, 44, 49–51)	Identification of protective and risk factors Determination of methods to mitigate/strengthen these risk/protective risks

Furthermore, women who are depressed and/or psychotic for suicide should be assessed for suicide as well. In presence of a SI or suicidal thought, clinicians should promptly developing a safety plan and referring to a specific psychiatric assessment as well as follow up the patient. In some case, hospitalization must be required as well. Urgency of referral depends on several factors including: whether SI is accompanied by a plan, whether there has been a history of suicide attempts, whether symptoms of a psychotic disorder are present. A risk assessment is helpful for identifying mothers at *low-risk* (SI or thought present, with a plan), *medium-risk* (SI with a plan or history of suicide attempt, without an immediate intent), or at *high-risk* (SI with an immediate intent). Warning signs of the risk of imminent suicide may include “feeling trapped,” “worthless, hopelessness, talking about death, writing a will, hoarding medication,” etc.

Suicide ideation is more likely associated with unplanned pregnancies, current mood and/or comorbid anxiety disorders, previous SI and/or suicidal attempt, and younger maternal age (9, 19, 21, 24, 26, 28–34).

Generally, screening during the perinatal period (particularly during pregnancy) represents an essential clinical tool for identifying women at higher risk of perinatal suicidality. Long-term identification and support of women at particular risk of maternal death due to suicide in the first year following birth may help lower the incidence of late maternal deaths. Overall, it should be proposed to clinicians, particularly gynecologists and primary care physicians, to ask to all pregnant women about their personal mental health history and family history. Women with a previous history of mental disorder (particularly, bipolar and MDDs as well as psychoses) should be offered a mental health assessment antenatally and managed by a psychiatrist. In addition, a regular interview on lifetime SI should be performed. Furthermore, mothers should be regularly monitored and supported for at least 12 months following delivery.

## Author Contributions

LO, AV, and CB conceived the topic of the manuscript, while LO, RV, and MF carried out the main analysis. CT and FI assisted in either screening of the studies or preparation of the attachments. DB, MP, and GP served as study reviewers. CB and GP served as senior study reviewers. All the coauthors substantially contributed to the present piece of work before approving it for final submission.

## Conflict of Interest Statement

The authors declare that the research was conducted in the absence of any commercial or financial relationships that could be construed as a potential conflict of interest.

## References

[1] Mathers CD Loncar D. Projections of global mortality and burden of disease from 2002 to 2030. PLoS Med (2006) 3(11):e.442. 10.1371/journal.pmed.0030442 PMC1664601 17132052

[2] Koslow SH Ruiz P Nemeroff CB A Concise Guide to Understanding Suicide. Cambridge: Cambrdige University Press (2014).

[3] Oquendo MA Halberstam B Mann JJ Risk factors for suicidal behavior: utility and limitations of research instruments. In: First MB, editor. Standardized Evaluation in Clinical Practice (2003). p. 103–30.

[4] Borges G Nock MK Haro Abad JM Hwang I Sampson NA Alonso J Twelve month prevalence of and risk factors for suicide attempts in the WHO World Mental Health Surveys. J Clin Psychiatry (2010) 71(12):1617 10.4088/JCP.08m04967blu 20816034 PMC3000886

[5] Brown GK Currier G Stanley B Suicide attempt registry pilot project. National Institute of Mental Health Annual Meeting of the Developing Centers for Intervention and Prevention of Suicide, September 2008. Canandaigua, NY (2008).

[6] Möller HJ Suicide, suicidality and suicide prevention in affective disorders. Acta Psychiatr Scand Suppl (2003) 108(418):73–80. 10.1034/j.1600-0447.108.s418.15.x 12956819

[7] Posner K Oquendo MA Gould M Stanley B Davies M Columbia Classification Algorithm of Suicide Assessment (C-CASA): classification of suicidal events in the FDA’s pediatric suicidal risk analysis of antidepressants. Am J Psychiatry (2007) 164(7):1035–43. 10.1176/ajp.2007.164.7.1035 17606655 PMC3804920

[8] Jones I Chandra PS Dazzan P Howard LM. Bipolar disorder, affective psychosis, and schizophrenia in pregnancy and the post-partum period. Lancet (2014) 384(9956):1789–99. 10.1016/S0140-6736(14)61278-2 25455249

[9] Newport DJ Levey LC Pennell PB Ragan K Stowe ZN. Suicidal ideation in pregnancy: assessment and clinical implications. Arch Womens Ment Health (2007) 10(5):181–7. 10.1007/s00737-007-0192-x 17726640

[10] Healey C Morriss R Henshaw C Wadoo O Sajjad A Scholefield H Self-harm in postpartum depression and referrals to a perinatal mental health team: an audit study. Arch Womens Ment Health (2013) 16(3):237–45. 10.1007/s00737-013-0335-1 23462983 PMC3661917

[11] Lindahl V Pearson JL Colpe L. Prevalence of suicidality during pregnancy and the postpartum. Arch Womens Ment Health (2005) 8(2):77–87. 10.1007/s00737-005-0080-1 15883651

[12] Copersino ML Jones H Tuten M Svikis D. Suicidal ideation among drug-dependent treatment-seeking inner-city pregnant women. J Maint Addict (2008) 3(2–4):53–64. 10.1300/J126v03n02_07 21796240 PMC3142928

[13] Palladino CL Singh V Campbell J Flynn H Gold KJ. Homicide and suicide during the perinatal period: findings from the National Violent Death Reporting System. Obstet Gynecol (2011) 118(5):1056–63. 10.1097/AOG.0b013e31823294da 22015873 PMC3428236

[14] Thornton C Schmied V Dennis CL Barnett B Dahlen HG. Maternal deaths in NSW (2000-2006) from nonmedical causes (suicide and trauma) in the first year following birth. Biomed Res Int (2013) 2013:623743. 10.1155/2013/623743 24024205 PMC3760299

[15] Fuhr DC Calvert C Ronsmans C Chandra PS Sikander S De Silva MJ Contribution of suicide and injuries to pregnancy-related mortality in low-income and middle-income countries: a systematic review and meta-analysis. Lancet Psychiatry (2014) 1(3):213–25. 10.1016/S2215-0366(14)70282-2 26360733 PMC4567698

[16] Do T Hu Z Otto J Rohrbeck P. Depression and suicidality during the postpartum period after first time deliveries, active component service women and dependent spouses, U.S. Armed Forces, 2007–2012. MSMR (2013) 20(9):2–7. 24093957

[17] Turkcapar AF Kadıoğlu N Aslan E Tunc S Zayıfoğlu M Mollamahmutoğlu L. Sociodemographic and clinical features of postpartum depression among Turkish women: a prospective study. BMC Pregnancy Childbirth (2015) 3(15):108. 10.1186/s12884-015-0532-1 25935726 PMC4491203

[18] Liberati A Altman DG Tetzlaff J Mulrow C Gøtzsche PC Ioannidis JP The PRISMA statement for reporting systematic reviews and meta-analyses of studies that evaluate health care interventions: explanation and elaboration. Ann Intern Med (2009) 151(4):W65–94. 10.7326/0003-4819-151-4-200908180-00136 19622512

[19] Khalifeh H Hunt IM Appleby L Howard LM. Suicide in perinatal and non-perinatal women in contact with psychiatric services: 15 year findings from a UK national inquiry. Lancet Psychiatry (2016) 3(3):233–42. 10.1016/S2215-0366(16)00003-1 26781366

[20] Johannsen BM Larsen JT Laursen TM Bergink V Meltzer-Brody S Munk-Olsen T. All-cause mortality in women with severe postpartum psychiatric disorders. Am J Psychiatry (2016) 173(6):635–42. 10.1176/appi.ajp.2015.14121510 26940804 PMC5325688

[21] Celik C Ozdemir B Oznur T Suicide risk among perinatal women who report thoughts of self-harm on depression screens. Obstet Gynecol (2015) 126(1):216–7. 10.1097/AOG.0000000000000941 26241284

[22] Esscher A Essén B Innala E Papadopoulos FC Skalidou A Sundström-Poromaa I Suicides during pregnancy and 1 year postpartum in Sweden, 1980–2007. Br J Psychiatry (2016) 208(5):462–9. 10.1192/bjp.bp.114.161711 26494874

[23] Pope CJ Xie B Sharma V Campbell MK. A prospective study of thoughts of self-harm and suicidal ideation during the postpartum period in women with mood disorders. Arch Womens Ment Health (2013) 16(6):483–8. 10.1007/s00737-013-0370-y 23784481

[24] Gavin AR Tabb KM Melville JL Guo Y Katon W. Prevalence and correlates of suicidal ideation during pregnancy. Arch Womens Ment Health (2011) 14(3):239–46. 10.1007/s00737-011-0207-5 21327844 PMC3724526

[25] Mauri M Oppo A Borri C Banti S PND-ReScU group. SUICIDALITY in the perinatal period: comparison of two self-report instruments. Results from PND-ReScU. Arch Womens Ment Health (2012) 15(1):39–47. 10.1007/s00737-011-0246-y 22215284

[26] Howard LM Flach C Mehay A Sharp D Tylee A. The prevalence of suicidal ideation identified by the Edinburgh Postnatal Depression Scale in postpartum women in primary care: findings from the RESPOND trial. BMC Pregnancy Childbirth (2011) 3(11):57. 10.1186/1471-2393-11-57 21812968 PMC3161003

[27] Schiff MA Grossman DC. Adverse perinatal outcomes and risk for postpartum suicide attempt in Washington state, 1987-2001. Pediatrics (2006) 118(3):e669–75. 10.1542/peds.2006-0116 16950958

[28] Kim JJ La Porte LM Saleh MP Allweiss S Adams MG Zhou Y Suicide risk among perinatal women who report thoughts of self-harm on depression screens. Obstet Gynecol (2015) 125(4):885–93. 10.1097/AOG.0000000000000718 25751206

[29] Sit D Luther J Buysse D Dills JL Eng H Okun M Suicidal ideation in depressed postpartum women: associations with childhood trauma, sleep disturbance and anxiety. J Psychiatr Res (2015) 6(6–67):95–104. 10.1016/j.jpsychires.2015.04.021 26001587 PMC4458196

[30] Huang H Faisal-Cury A Chan YF Tabb K Katon W Menezes PR. Suicidal ideation during pregnancy: prevalence and associated factors among low-income women in São Paulo, Brazil. Arch Womens Ment Health (2012) 15(2):135–8. 10.1007/s00737-012-0263-5 22382280 PMC3727148

[31] Tavares D Quevedo L Jansen K Souza L Pinheiro R Silva R. Prevalence of suicide risk and comorbidities in postpartum women in Pelotas. Rev Bras Psiquiatr (2012) 34(3):270–6. 10.1016/j.rbp.2011.12.001 23429772

[32] Farias DR Pinto Tde J Teofilo MM Vilela AA Vaz Jdos S Nardi AE Prevalence of psychiatric disorders in the first trimester of pregnancy and factors associated with current suicide risk. Psychiatry Res (2013) 210(3):962–8. 10.1016/j.psychres.2013.08.053 24090486

[33] Chen YH Lau G Maternal deaths from suicide in Singapore. Singapore Med J (2008) 49(9):694–7. 18830543

[34] Comtois KA Schiff MA Grossman DC Psychiatric risk factors associated with postpartum suicide attempt in Washington State, 1992–2001. Am J Obstet Gynecol (2008) 199:120.e1–5. 10.1016/j.ajog.2008.02.011 18355781

[35] Alhusen JL Frohman N Purcell G. Intimate partner violence and suicidal ideation in pregnant women. Arch Womens Ment Health (2015) 18(4):573–8. 10.1007/s00737-015-0515-2 25753680 PMC4506210

[36] Stewart DE The importance of intimate partner violence and suicidal ideation in pregnant women. Arch Womens Ment Health (2015) 18(4):571–2. 10.1007/s00737-015-0539-7 25981973

[37] Fisher J Tran TD Biggs B Dang TH Nguyen TT Tran T. Intimate partner violence and perinatal common mental disorders among women in rural Vietnam. Int Health (2013) 5(1):29–37. 10.1093/inthealth/ihs012 24029843

[38] Coelho FM Pinheiro RT Silva RA de Ávila Quevedo L de Mattos Souza LD de Matos MB Parental bonding and suicidality in pregnant teenagers: a population-based study in southern Brazil. Soc Psychiatry Psychiatr Epidemiol (2014) 49(8):1241–8. 10.1007/s00127-014-0832-1 24562317

[39] Vaz JS Kac G Nardi AE Hibbeln JR. Omega-6 fatty acids and greater likelihood of suicide risk and major depression in early pregnancy. J Affect Disord (2014) 15(2–154):76–82. 10.1016/j.jad.2013.04.045 23726775 PMC4239694

[40] Appleby L Mortensen PB Faragher EB. Suicide and other causes of mortality after post-partum psychiatric admission. Br J Psychiatry (1998) 173:209–11. 10.1192/bjp.173.3.209 9926095

[41] Oates M. Perinatal psychiatric disorders: a leading cause of maternal morbidity and mortality. Br Med Bull (2003) 67:219–29. 10.1093/bmb/ldg011 14711766

[42] Castro e Couto T Brancaglion MY Cardoso MN Faria GC Garcia FD Nicolato R Suicidality among pregnant women in Brazil: prevalence and risk factors. Arch Womens Ment Health (2016) 19(2):343–8. 10.1007/s00737-015-0552-x 26189445

[43] Gelaye B Barrios YV Zhong QY Rondon MB Borba CP Sánchez SE Association of poor subjective sleep quality with suicidal ideation among pregnant Peruvian women. Gen Hosp Psychiatry (2015) 37(5):441–7. 10.1016/j.genhosppsych.2015.04.014 25983188 PMC4558240

[44] Girardi P Pompili M Innamorati M Serafini G Berrettoni C Angeletti G Temperament post-partum depression, hopelessness, and suicide risk among women soon after delivering. Women Health (2011) 51(5):511–24. 10.1080/03630242.2011.583980 21797682

[45] Einarson A Selby P Koren G. Abrupt discontinuation of psychotropic drugs during pregnancy: fear of teratogenic risk and impact of counselling. J Psychiatry Neurosci (2001) 26(1):44–8. 11212593 PMC1408034

[46] Pinheiro RT de Silva RA Magalhães PV Horta BL Pinheiro KA Two studies on suicidality in the postpartum. Acta Psychiatr Scand (2008) 118(2):160–3. 10.1111/j.1600-0447.2008.01184.x 18498435

[47] Turner LA Kramer MS Liu S Maternal mortality and morbidity study group of the Canadian perinatal surveillance system. Cause-specific mortality during and after pregnancy and the definition of maternal death. Chronic Dis Can (2002) 23(1):31–6. 11876834

[48] Mundt JC Greist JH Jefferson JW Federico M Mann JJ Posner K Prediction of suicidal behaviour in clinical research by lifetime suicidal ideation and behavior ascertained by the electronic Columbia-Suicide Severity Rating Scale. J Clin Psychiatry (2013) 74(9):887–93. 10.4088/JCP.13m08398 24107762

[49] Hamilton M A rating scale for depression. J Neurol Neurosurg Psychiatry (1960) 23:56–62. 10.1136/jnnp.23.1.56 14399272 PMC495331

[50] Beck AT Ward CH Mendelson M Mock J Erbaugh J An inventory for measuring depression. Arch Gen Psychiatry (1961) 4:561–71. 10.1001/archpsyc.1961.01710120031004 13688369

[51] Cox JL Holden JM Sagovsky R. Detection of postnatal depression. Development of the 10-item Edinburgh Postnatal Depression Scale. Br J Psychiatry (1987) 150:782–6. 10.1192/bjp.150.6.782 3651732

[52] Kroenke K Spitzer RL Williams JB. The PHQ-9: validity of a brief depression severity measure. J Gen Intern Med (2001) 16(9):606–13. 10.1046/j.1525-1497.2001.016009606.x 11556941 PMC1495268

[53] Gibson J McKenzie-McHarg K Shakespeare J Price J Gray R. A systematic review of studies validating the Edinburgh Postnatal Depression Scale in antepartum and postpartum women. Acta Psychiatr Scand (2009) 119(5):350–64. 10.1111/j.1600-0447.2009.01363.x 19298573

[54] Matthey S. Using the Edinburgh Postnatal Depression Scale to screen for anxiety disorders. Depress Anxiety (2008) 25(11):926–31. 10.1002/da.20415 18041072

[55] Zhong QY Gelaye B Rondon MB Sánchez SE Simon GE Henderson DC Using the Patient Health Questionnaire (PHQ-9) and the Edinburgh Postnatal Depression Scale (EPDS) to assess suicidal ideation among pregnant women in Lima, Peru. Arch Womens Ment Health (2015) 18(6):783–92. 10.1007/s00737-014-0481-0 25369907 PMC4635023

[56] Williams JA. A structured interview guide for the Hamilton Depression Rating Scale. Arch Gen Psychiatry (1988) 45:742–7. 10.1001/archpsyc.1988.01800320058007 3395203

